# Editorial: Public health, public health education, and their future prospects

**DOI:** 10.3389/fpubh.2024.1403535

**Published:** 2024-04-04

**Authors:** Yusha Wang, Yihui Du, Hubing Shi, Xuelei Ma

**Affiliations:** ^1^Department of Biotherapy, Cancer Center and State Key Laboratory of Biotherapy, West China Hospital, Sichuan University, Chengdu, Sichuan, China; ^2^Lung Cancer Center, West China Hospital, Sichuan University, Chengdu, Sichuan, China; ^3^Department of Epidemiology, University Medical Center Groningen, University of Groningen, Groningen, Netherlands

**Keywords:** public health, public health education, editorial, education mode, COVID-19

Based on the need of healthcare worker with self-learning potential and public health skill, the medical education mode has been constantly explored and reformed. Novel teaching modes such as lecture-based learning (LBL), game-based learning (GBL), and team-based learning (TBL), empower students to learn and grow better compared with conventional instructor-led mode (1–3). Due to the rising awareness of public health, medical teaching mode has been momentous facilitated in the epidemic era. Therefore, this Research Topic recruited studies including multiple approaches for education, impact of COVID-19 epidemic on the public health, and researches on other diseases which serve as a public burden.

In recent years, with the gradual enrichment of non-traditional education strategies, education models have been widely discussed and applied. In this Research Topic, some articles explored different novel education modes. For instance, Zhang et al. systematically reviewed randomized controlled trials about TBL and LBL in nursing education, and found that TBL was generally more effective than LBL in improving academic achievement and overall competence. Xu et al. reviewed recent researches on the GBL in medical education, and found that serious games and gamification could improve the participation of students in class, and stimulate the motivation to learn. In Saudi Arabian, Alrabiah et al. investigated pharmacy students' perceptions of arithmetic and their mathematical abilities, thus found that integrating mathematics focused instruction into pharmacy curricula could improve its applicability in healthcare. Moreover, blended learning which refers to the combination of face-to-face teaching and online learning, has become increasingly popular. Study of Wang C. et al. showed that the inclusion of online learning tools in physical education curricula had a significant benefit, whereas instructional design, technological literacy, and self-regulation strategies are needed.

Owing to the COVID-19 epidemic, the application of online distance learning mode is widespread around the world. Qiu et al. studied the effect of online distance and on-site teaching modes. The general teaching effect was proven to be comparable, while domestic on-site teaching mode may provide better in-class teaching effects. With the modern technology rapid development and the educational mode iteration, Wang X. et al. explored the ability of artificial intelligence (AI) to process massive data, the value to provide personalized and efficient educational solution, and the potential to revolutionize public health education. Additionally, Qu et al. found a correlation between procrastination and burnout among medical students during the epidemic in China, with both positively related to negative academic sentiment. Consequently, the role of general academic emotions (GAEs) in procrastination and burnout was emphasized. Notably, during the online teaching mode rising application worldwide, digital sobriety strategy is believed to reduce carbon emissions and enhance the sustainable development of digital technology. Gandhi et al. found that the carbon footprint of continuing medical education (CME) was significantly lower under the virtual digital sobering strategy in India, but the satisfaction of virtual learning was low. The aforementioned articles explored different teaching methods, suggesting that there is hope for reforming medical education modes and applying them to practice.

Health literacy plays an essential role in this era as the public becomes more reliant on digital resources. Accordingly, there are articles in this Research Topic that focus on health literacy and attempt to establish health literacy assessment tool, as well as explore the quality of evidence in public health research. Lau et al. assessed the COVID-19 specific health literacy of teachers in Hong Kong. Results showed that about half of teachers possessed adequate health literacy, while a large proportion of teachers still show poor understanding of health. It is highlighted that interventions should be carried out to improve health literacy of teachers. A questionnaire for the assessment of health information literacy level developed by Yu et al., and the results demonstrated that it was a valuable tool with good reliability and validity. Furthermore, Xun et al. systematically explored the differences in the sources of evidence, the types of primary studies, and the tools evaluating the quality of studies in public health systematic reviews (SRs). The results indicated that SRs should evaluate the quality of individual studies and evidence for these results.

Not only that, this Research Topic included some articles on other public health issues. Situational awareness (SA) is the basis of decision-making process and an important cognitive construct for positive safety outcomes of patients. Ghaderi et al. have shown that the SA of ICU nurses includes perceiving the cues of patient and environment, understanding the current situation by analyzing cues, and predicting the state of patients. The risk of disability connected with health-related factors and chronic diseases is studied in a wide range. Kim et al. found that factors such as smoking, alcohol consumption, and physical activity were associated with new disabilities, while physical activity and people with higher education and income levels were at low risk. These results can be a vital foundation for health care policies and strategies to prevent and reduce disability. The incidence of AIDS is substantially increasing year by year, and effective prevention is necessary. Bahikire et al. investigated the low use rate of non-occupational post-exposure prophylaxis (nPEP) to prevent HIV acquisition among fishers in Uganda, was mainly by virtue of lacking nPEP awareness. Thereby, increased community sensitization and improved HIV prevention education, are important. Walking is recognized and considered to be the most convenient form of physical activity (PA). Safi et al. aimed to promote PA for university employees by recommending daily steps and providing incentives on a team basis. This rewarding intervention had a positive impact on the personal and work outcomes.

As for the management of diabetes, Pi et al. found significant gaps in knowledge and management practices among primary care providers (PCPs) in the Central China region, highlighting the need of structured programs to enhance the prediabetes-related knowledge and practice of PCPs. It is important to formulate intervention strategies about physical activity in coronary heart disease (CHD) patients. Alsaleh and Baniyasin discussed the low level of physical activity in CHD patients in Jordan, and identified the barriers incorporating psychological, environmental, and health-related factors. Likewise, Guo Z. et al. investigated that the majority in South China had a moderate perception of cardiovascular disease risk, whereas individuals with high blood pressure, alcohol consumption, and better subjective health status underestimate the risk. And older age, higher income, diabetes, and better health are greatly associated with a higher cardiovascular disease risk. Metabolic syndrome (MetS) is known as a constellation of metabolic disorders comprising dyslipidemia, increased arterial blood pressure, and impaired fasting glycemia. Guo L. et al. found that more than 11 h per day for sedentary time was correlated well with an increased risk of MetS among Tibetans in China. These findings emphasized the importance of health education and reducing sedentary time.

In conclusion, articles in this Research Topic provide valuable insights and guidance for medical education modes and public health issues in the epidemic era. Among these articles, teaching modes such as TBL, LBL, and GBL contribute to learn basic medical knowledge efficiently for medical students. Online distance and AI assisted teaching modes promote to complete GME and obtain cutting-edge medical information for medical staff. Moreover, articles that addressed the causes, prevention, and management of other public health problems such as AIDS, diabetes, and CHD, are able to reduce the public health burden.

## Author contributions

YW: Writing – original draft. YD: Writing – review & editing. HS: Writing – review & editing. XM: Writing – review & editing.
